# Intensity-modulated radiation therapy achieves better local control compared to three-dimensional conformal radiation therapy for T4-stage nasopharyngeal carcinoma

**DOI:** 10.18632/oncotarget.12736

**Published:** 2016-10-18

**Authors:** Jenny Ling-Yu Chen, Yu-Sen Huang, Sung-Hsin Kuo, Ruey-Long Hong, Jenq-Yuh Ko, Pei-Jen Lou, Chun-Wei Wang

**Affiliations:** ^1^ Department of Oncology, National Taiwan University Hospital and National Taiwan University College of Medicine, Taipei, Taiwan; ^2^ Department of Radiation Oncology, National Taiwan University Hospital Hsin-Chu Branch, Hsin-Chu, Taiwan; ^3^ Department of Medical Imaging, National Taiwan University Hospital and National Taiwan University College of Medicine, Taipei, Taiwan; ^4^ Department of Medical Imaging, National Taiwan University Hospital Yun-Lin Branch, Yun-Lin, Taiwan; ^5^ Graduate Institute of Oncology, National Taiwan University, Taipei, Taiwan; ^6^ Department of Otolaryngology, National Taiwan University Hospital and National Taiwan University College of Medicine, Taipei, Taiwan

**Keywords:** intensity-modulated radiation therapy, conformal radiation therapy, nasopharyngeal carcinoma, survival outcomes, late toxicities

## Abstract

**Purpose:**

To examine the survival outcomes and late toxicity profiles of three-dimensional conformal radiation therapy (3DCRT) *vs*. intensity-modulated radiation therapy (IMRT) for patients with nasopharyngeal carcinoma (NPC).

**Methods:**

Three hundred and seventy-four patients with newly diagnosed, non-metastatic, NPC who were curatively treated with 3DCRT between 2004 and 2006 and 481 patients treated with IMRT between 2007 and 2009 were analyzed. Patients were categorized as having advanced-stage disease (stage III, IVA, and IVB disease; *n* = 709) or early-stage disease (stage I and II; *n* = 146). The median follow-up time was 90.3 months for patients treated with 3DCRT and 86.3 months for patients treated with IMRT.

**Results:**

For early-stage patients, the outcomes of IMRT *vs*. 3DCRT were similar considering locoregional control (LRC), distant metastasis-free survival (DMFS), and overall survival (OS). For advanced-stage patients, IMRT was associated with better LRC compared with 3DCRT (5-year LRC rate: 85.6% *vs*. 76.6%, respectively; *p* = 0.035) and OS (5-year OS rate: 82.3% *vs*. 71.8%, respectively; *p* = 0.002), whereas DMFS was similar for both treatments (5-year DMFS rate: 80.9% *vs*. 79.0%, respectively; *p* = 0.324). Furthermore, the IMRT technique was more beneficial for patients with T4 disease. Late toxicities occurred more frequently in patients treated with 3DCRT than in those treated with IMRT (grade ≥3 neck fibrosis: 6.7% *vs*. 3.7%, respectively, *p* = 0.036; radiographic temporal lobe necrosis: 10.2% *vs*. 4.4%, respectively, *p* < 0.001).

**Conclusions:**

Compared with 3DCRT, IMRT offered better LRC in patients with advanced-stage non-metastatic NPC, which corresponded with better OS.

## INTRODUCTION

Nasopharyngeal carcinoma (NPC) is a highly radiosensitive tumor, and definitive radiation therapy (RT) is the standard treatment for it [1, 2]. Modern RT techniques have emerged with the development of conformal RT (CRT), such as three-dimensional CRT (3DCRT) or intensity-modulated RT (IMRT). CRT allows better delineation of the tumor target and organs at risk with clearer radiologic visualization of their spatial relations, thus providing a potential therapeutic benefit of dose escalation to tumor tissues, with reduced toxicity to normal tissues.

IMRT achieves better dose differentiation between tumorous and normal tissues compared with 3DCRT and facilitates simultaneous delivery of different fractional doses to different targets [3, 4]. In addition, IMRT has the advantage of better tumor coverage because it allows for dose escalation, while reducing exposure to the parotid gland, temporomandibular joints, and brainstem/temporal lobe. Thus, IMRT is superior to 3DCRT in that it increases the biologic effect on the tumor owing to physical dose escalation, while avoiding toxicity to critical tissues [5, 6].

The technical and dosimetric superiority of RT techniques could translate into clinical benefits, such as reduced normal tissue toxicity, improved quality of life, and increased locoregional control (LRC) and survival outcomes [7–11]. Despite the potential advantages of IMRT for NPC, the exact survival benefit and late toxicity reduction after treatment with IMRT compared with 3DCRT are not known; in addition, there is no evidence about which patient population would most benefit from IMRT treatment. The study aimed to compare the survival outcomes and late toxicity profiles after treatment with 3DCRT and IMRT for patients with NPC.

## RESULTS

### Patient characteristics

A total of 855 NPC patients who were treated with curative intent with 3DCRT (*n* = 374) between 2004 and 2006 or with IMRT (*n* = 481) between 2007 and 2009 were analyzed. Patient and treatment characteristics are listed in Table 1. The median patient age was 49 years (range, 10-93 years). Four patients (2 in the 3DCRT group and 2 in the IMRT group) developed life-threatening sepsis and expired before completing radiotherapy. These four patients had a follow-up time of 0 months. The median follow-up time was 90.3 months (range, 0-111.2 months) for patients treated with 3DCRT and 86.3 months (range, 0-111.4 months) for patients treated with IMRT. As shown in Table 1, compared with the patients in the 3DCRT group, patients in the IMRT group were diagnosed at earlier T and N stages (*p* < 0.001 and *p* = 0.017, respectively). Advanced-stage disease (stage III, IVA, and IVB) was more commonly observed in the 3DCRT group (92.0% *vs*. 75.9% in the IMRT group, *p* < 0.001). These chronologic epidemiologic data suggest the implementation of nasopharyngeal screening during clinical practice. Of the 349 patients in the 3DCRT group and 452 patients in the IMRT group who had concurrent systemic therapy, 32 patients and 29 patients, respectively, received 100 mg/m^2^ cisplatin every 3 weeks for 2-3 cycles, while the remaining patients received 30 mg/m^2^ cisplatin every week.

**Table 1:** Patient clinical characteristics and treatment parameters

		3DCRT (*n* = 374)		IMRT (*n* = 481)		*P*-value
		*n*	%	*n*	%	
Age						0.361
	≤60 years	302	80.7	400	83.2	
	>60 years	72	19.3	81	16.8	
Sex						0.149
	Male	265	70.9	362	75.3	
	Female	109	29.1	119	24.7	
T stage						<0.001*
	T1 or T2	76	20.3	207	43.0	
	T3 or T4	298	79.7	274	57.0	
N stage						0.017*
	≤N2	277	74.1	389	80.9	
	N3	97	25.9	92	19.1	
Early *vs*. Advanced stage						<0.001*
	Early (stage I and II)	30	8.0	116	24.1	
	Advanced (stage III, IVA, and IVB)	344	92.0	365	75.9	
Duration						0.793
	≤50 days	183	48.9	231	48.0	
	>50 days	191	51.1	250	52.0	
Induction therapy						<0.001*
	No	231	61.8	384	79.8	
	Yes	143	38.2	97	20.2	
Concurrent systemic therapy						0.696
	No	25	6.7	29	6.0	
	Yes	349	93.3	452	94.0	

*Abbreviations*: 3DCRT = three-dimensional conformal radiation therapy; IMRT = intensity-modulated radiation therapy. *P*-values obtained by using the Student *t*-test. *P < 0.05 for comparison between 3DCRT and IMRT.

### Treatment outcomes

In the overall population, the median LRC, DMFS, and OS were 79.2 months, 81.7 months, and 83.0 months, respectively. At the time of analysis, 301 patients (35.2%) had recurrence at ≥1 sites, and 192 (22.5%) had died of the disease. Univariate and multivariate analyses (Table 2) revealed that old age, advanced stage, and use of the 3DCRT technique were associated with poor outcomes. No significant difference was found for the different concurrent chemotherapy regimens (100 mg/m^2^ cisplatin every 3 weeks or 30 mg/m^2^ cisplatin every week) on LRC (*p* = 0.563), DMFS (*p* = 0.090), or OS (*p* = 0.294).

**Table 2:** Univariate and multivariate analyses of potential prognostic factors

**Univariate analysis**									
			**LRC**			**DMFS**		**OS**	
			***P*-value**			***P*-value**		***P*-value**	
Age (≥60 *vs*. <60 years)			0.074			0.006*		<0.001*	
Sex (M *vs*. F)			0.826			0.071		0.265	
Early *vs*. Advanced stage			<0.001*			<0.001*		<0.001*	
3DCRT *vs*. IMRT			0.007*			0.047*		<0.001*	
RT duration (>50 *vs*. ≤50 days)			0.567			0.316		0.272	
Induction therapy (No *vs*. yes)			0.919			0.070		0.786	
Concurrent systemic therapy (No *vs*. yes)			0.797			0.064		0.911	
**Multivariate analysis**									
	**LRC**			**DMFS**			**OS**		
	**HR (95% CI)**	***P*-value**		**HR (95% CI)**	***P*-value**		**HR (95% CI)**		***P*-value**
Age (≥60 *vs*. <60 years)	1.4 (0.9-2.1)	0.076		1.7 (1.1-2.4)	0.009*		3.0 (2.2-4.0)		<0.001*
Early *vs*. Advanced stage	1.9 (1.3-2.8)	0.002*		2.5 (1.6-3.9)	<0.001*		2.1 (1.4-3.0)		<0.001*
3DCRT *vs*. IMRT	1.4 (1.1-1.9)	0.014*		1.1 (0.8-1.6)	0.439		1.5 (1.1-2.0)		0.005*

*Abbreviations*: LRC = locoregional control; DMFS = distant metastasis-free survival; OS = overall survival; HR = hazard ratio; CI = confidence interval; 3DCRT = three-dimensional conformal radiation therapy; IMRT = intensity-modulated radiotherapy; * *P* < 0.05 with statistical significance.

**Figure 1:**
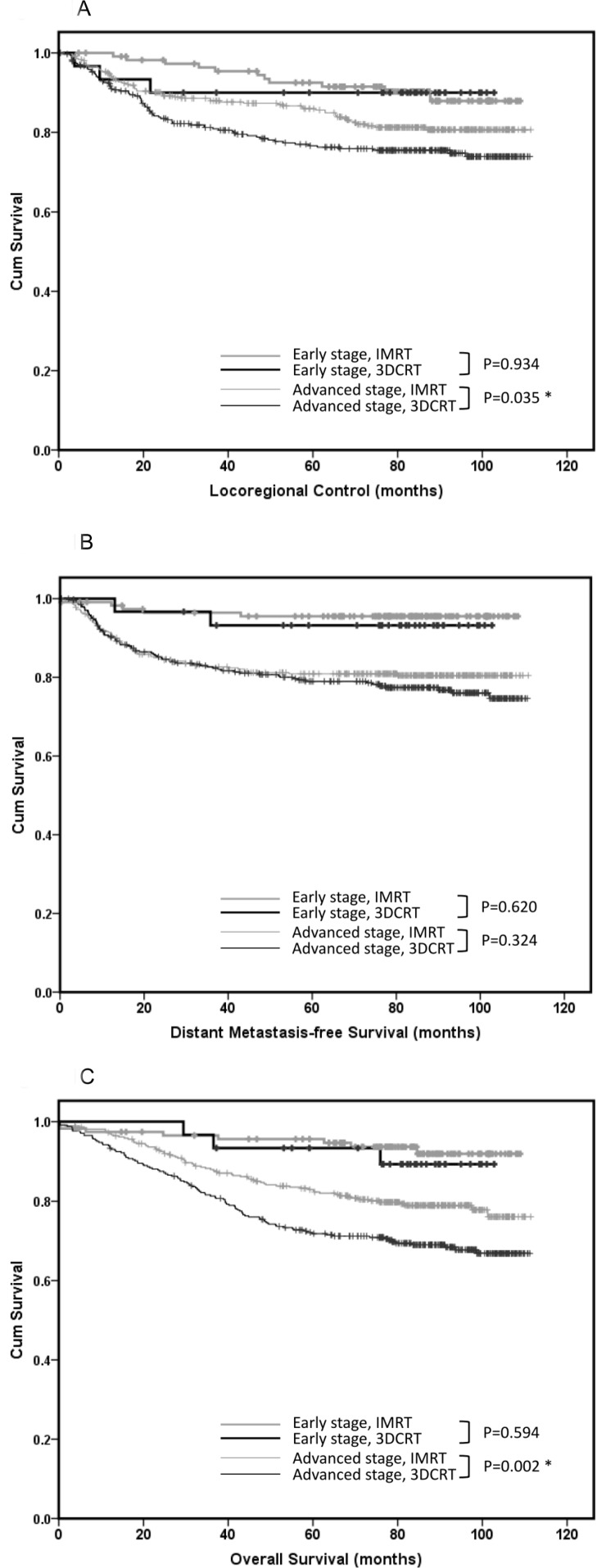
Outcomes of patients with early and advanced-stage nasopharyngeal carcinoma The outcomes of patients with early and advanced-stage nasopharyngeal carcinoma treated with three-dimensional conformal radiation therapy (3DCRT, *n* = 374) versus those treated with intensity-modulated radiation therapy (IMRT, *n* = 481) considering **A**. locoregional control, **B**. distant metastasis-free survival, and **C**. overall survival.

**Figure 2:**
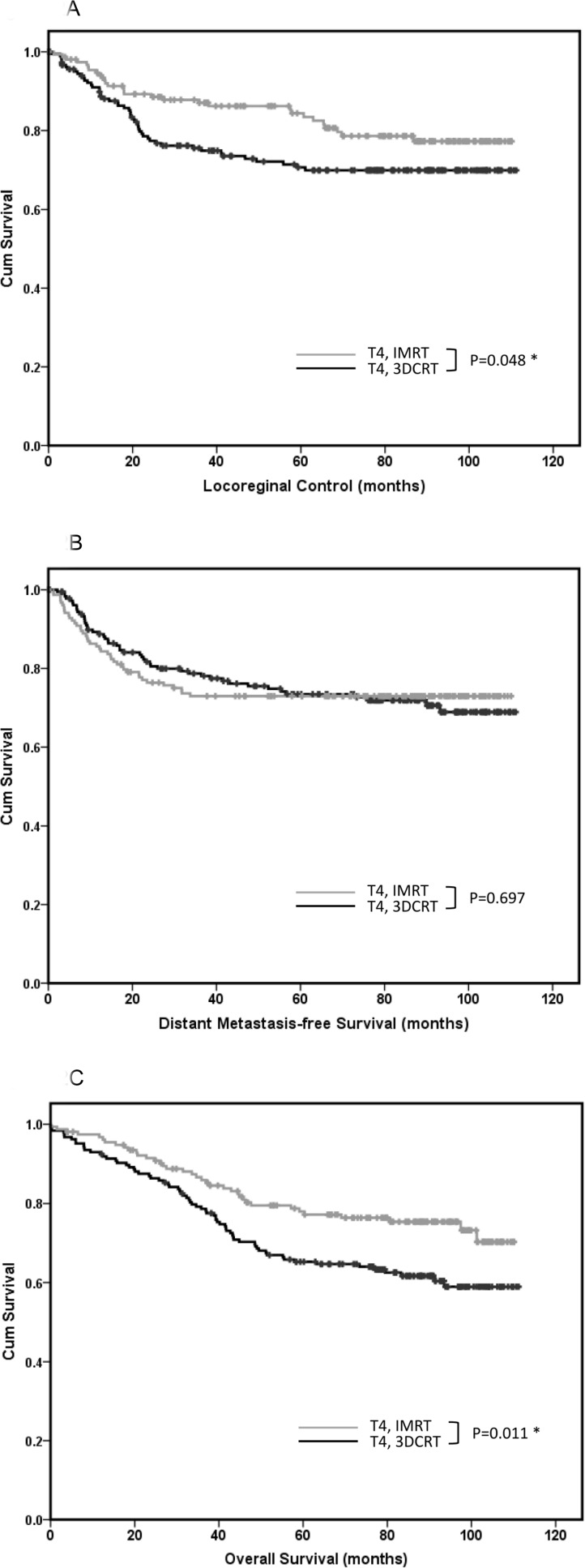
Outcomes of patients with T4 nasopharyngeal carcinoma The outcomes of patients with T4 nasopharyngeal carcinoma treated with three-dimensional conformal radiation therapy (3DCRT, *n* = 184) versus those treated with intensity-modulated radiation therapy (IMRT, *n* = 154) considering **A**. locoregional control, **B**. distant metastasis-free survival, and **C**. overall survival.

To eliminate the selection bias of more advanced-stage patients in the 3DCRT group that would have led to poor survival outcomes, early and advanced-stage patients were separated to compare the two radiation therapy techniques. As shown in Figure 1, for early-stage patients, the outcomes of IMRT and 3DCRT were similar considering the LRC (LRC, 5-year LRC rate: 92.5% *vs*. 90.0%, respectively; *p* = 0.934), DMFS (DMFS, 5-year DMFS rate: 95.5% *vs*. 93.2%, respectively; *p* = 0.620), and OS (OS, 5-year OS rate: 95.6% *vs*. 89.3%, respectively; *p* = 0.594). For advanced-stage patients, IMRT, compared with 3DCRT, was associated with better LRC (5-year LRC rate: 85.6% *vs*. 76.6%, respectively; *p* = 0.035) and OS (5-year OS rate: 82.3% *vs*. 71.8%, respectively; *p* = 0.002), whereas DMFS was similar for both the treatments (5-year DMFS rate: 80.9% *vs*. 79.0%, respectively; *p* = 0.324). To determine which subgroup of advanced stage cancer would benefit the most from IMRT and 3DCRT, the survival outcomes were compared. As shown in Table 3, the IMRT technique was more beneficial for patients with T4 disease than for those with T3, N2, or N3 disease. In advanced-stage patients with T4 disease, IMRT was associated with better LRC, compared to 3DCRT (5-year LRC rate: 83.4% *vs*. 70.6%, respectively; *p* = 0.048) and OS (5-year OS rate: 77.2% *vs*. 65.3%, respectively; *p* = 0.011; Figure 2), whereas DMFS was similar for both treatments (5-year DMFS rate: 73.0% *vs*. 73.5%, respectively; *p* = 0.697).

**Table 3:** Survival outcomes of advanced-stage patients treated with 3DCRT *vs*. IMRT

	5-year survival rate	3DCRT	IMRT	*P*-value
T3 (*n* = 234)		*n* = 114	*n* = 120	
	LRC	82.2%	86.5%	0.379
	DMFS	87.2%	85.2%	0.952
	OS	79.7%	86.3%	0.562
T4 (*n* = 338)		*n* = 184	*n* = 154	
	LRC	70.6%	83.4%	0.048*
	DMFS	73.5%	73.0%	0.697
	OS	65.3%	77.2%	0.011*
N2 (*n* = 362)		*n* = 184	*n* = 178	
	LRC	81.1	88.7	0.312
	DMFS	83.4	84.4	0.597
	OS	76.9	87.1	0.044*
N3 (*n* = 189)		*n* = 97	*n* = 92	
	LRC	71.8%	82.6%	0.068
	DMFS	65.4%	71.4%	0.274
	OS	60.6%	71.7%	0.096

*Abbreviations*: LRC = locoregional control; DMFS = distant metastasis-free survival; OS = overall survival; 3DCRT = three-dimensional conformal radiation therapy; IMRT = intensity-modulated radiation therapy. *P*-values obtained by using Kaplan-Meier life table analysis and the log-rank test. * *P* < 0.05 for comparison between 3DCRT and IMRT.

### Radiation therapy late toxicity

As shown in Table 4, grade ≥3 toxicities occurred in 27.8% and 27.5% of patients treated with IMRT and 3DCRT, respectively (*p* = 0.787). No significant difference was seen considering grade ≥3 ototoxicity, cranial neuropathy, xerostomia, or osteoradionecrosis. In the IMRT and 3DCRT groups, 54 patients (11.2%) and 46 (12.3%), respectively, experienced otologic toxicities and required tympanocentesis, grommet insertion, myringotomy, or hearing aids; 38 (7.9%) and 30 (8.0%) developed transient or permanent cranial neuropathy not attributed to locoregional recurrence, and they required hospitalization or more aggressive treatment; 13 (2.7%) and 12 (3.2%) had salivary gland dysfunction without any response to drug stimulation; and 19 (3.9%) and 11 (2.9%) patients experienced osteonecrosis necessitating medication, hyperbaric oxygen, or operative intervention. In total, 18 patients (3.7%) in the IMRT group versus 25 (6.7%) in the 3DCRT group experienced soft tissue toxicities with severe induration and loss of subcutaneous tissue, thereby requiring aggressive rehabilitation (*p* = 0.036). Radiographic temporal lobe necrosis occurred more frequently in patients treated with 3DCRT than in those treated with IMRT (10.2% *vs*. 4.4%, respectively; *p* < 0.001).

**Table 4:** Radiation therapy late toxicities in patients treated with 3DCRT *vs*. IMRT

	3DCRT (*n* = 374)			IMRT (*n* = 481)			*P*-value
	Grade 3 (%)	Grade 4 (%)	Grade 5 (%)	Grade 3 (%)	Grade 4 (%)	Grade 5 (%)	
Otitis/hearing loss	46 (12.3%)	0	0	54 (11.2%)	0	0	0.594
Soft tissue fibrosis	24 (6.4%)	1 (0.3%)	0	17 (3.5%)	1 (0.2%)	0	0.036*
Cranial neuropathy	27 (7.2%)	3 (0.8%)	0	38 (7.9%)	0	0	0.948
Salivary gland dysfunction	12 (3.2%)	0	0	13 (2.7%)	0	0	0.373
Osteonecrosis	8 (2.1%)	3 (0.8%)	0	18 (3.7%)	1 (0.2%)	0	0.426
Epistaxis bleeding	3 (0.8%)	7 (1.9%)	2 (0.5%)	2 (0.4%)	8 (1.7%)	2 (0.4%)	0.531
Any late toxicities	86 (23.0%)	15 (4.0%)	2 (0.5%)	120 (24.9%)	12 (2.5%)	2 (0.4%)	0.787
Radiographic temporal lobe necrosis	38 (10.2%)			21 (4.4%)			<0.001*

*Abbreviations*: 3DCRT = three-dimensional conformal radiation therapy; IMRT = intensity-modulated radiation therapy. *P*-values obtained by using the Pearson chi-square test. * *P* < 0.05 for the comparison of ≥Grade 3 toxicities between 3DCRT and IMRT.

## DISCUSSION

The current study—a large series that focused on patients with non-metastatic NPC treated using 3DCRT or IMRT—was successful in determining the efficacy of modern radiation techniques considering the survival outcomes and late toxicities in patients treated with NPC. Although several studies have demonstrated that IMRT has better dose distributions compared with 3DCRT, the clinical outcome of increasing LRC is still under investigation [7–12]. A retrospective study had shown the benefit of IMRT over two-dimensional radiation therapy (2DRT) considering LRC in early T-stage patients [9]; however, other studies comparing IMRT and 2DRT or 3DCRT had shown the benefit of IMRT for LRC in advanced patients [10, 13], which is in line with our findings. IMRT planning has the advantage of achieving steeper dose gradients between tumorous and normal tissues, and thus, IMRT results in good LRC in patients with T4 NPC [3, 4].

In our series, IMRT reduced the frequency of late toxicities of RT, including severe neck fibrosis and radiographic temporal lobe necrosis, which is in line with the findings of several retrospective studies that revealed a better quality of life after IMRT compared with that obtained after 3DCRT [7, 8, 11]. Other studies comparing IMRT and 2DRT have also shown decreased temporal lobe injury, cranial neuropathy, trismus, neck fibrosis, xerostomia, or hearing loss in the IMRT group [9–11, 14].

Our study has several limitations, including the retrospective design. The patient population included was heterogeneous considering the stage, induction chemotherapy, concurrent chemotherapy regimens, and multimodality treatments. Efforts were made to obtain equivalent follow-up times in the different radiotherapy groups, in order to eliminate bias related to timing. The plasma Epstein-Barr virus (EBV) DNA measurement has been incorporated into standard medical practice in several medical centers to help diagnose, monitor, and predict NPC patients’ survival outcomes [15–17]. Our institution has obtained EBV DNA measurements since 2014; therefore, no comprehensive data were available regarding EBV DNA measurements for the patient cohort in this study, which retrospectively included patients from 2004 to 2009. We plan to incorporate these data in future clinical investigations, gaining valuable insights.

IMRT is regarded as the standard care for NPC patients in most developed countries. Owing to the uneven distribution of economic medical resources, a small number of patients are still being treated with 3DCRT technique in the modern era. Our results showed that 3DCRT achieved equivalent treatment outcomes for early-stage patients, compared to IMRT; however, 3DCRT might not be as effective for improving advanced-stage patients LRC, compared to IMRT, indicating that 3DCRT might be sufficient for early-stage patients, while IMRT is ideal for T4 or advanced-stage patients. Furthermore, when economical resources are limited, choosing 3DCRT for early-stage patients and IMRT for advanced-stage patients might be the optimal approach. More evidence is needed to enhance the continuous and comprehensive evolution of radiation therapy, bringing improved approaches and outcomes. We believe that our study may provide a modest contribution to the basis of modern radiation treatment, with emphasis on refinement and precision.

In conclusion, the results of our study suggest that IMRT offered good LRC in patients with advanced-stage non-metastatic NPC, especially in T4 patients, which corresponded with better OS.

## MATERIALS AND METHODS

### Patients

This study was approved by the Institutional Review Board of our institute (201310016RINA). As per institutional policy, patients with NPC were treated with IMRT instead of 3DCRT since January 2007. Accordingly, 374 patients with newly diagnosed, non-metastatic NPC who were curatively treated with 3DCRT between 2004 and 2006 and 481 patients treated with IMRT between 2007 and 2009 were analyzed. According to the American Joint Commission on Cancer classification system, seventh edition [18], patients were categorized as having advanced-stage disease (stage III, IVA, or IVB disease; *n* = 709) or early-stage disease (stage I and II; *n* = 146). Staging evaluations included magnetic resonance imaging (MRI) of the nasopharynx and neck, chest radiography, liver ultrasonography, and bone scintigraphy, as well as positron emission tomography/computed tomography (CT), if clinically required.

### Radiation therapy

The RT treatment technique used to treat the patients evaluated in this study has been previously described [1, 4, 5]. Briefly, a thermoplastic mask was used to ensure immobilization of the head to the shoulder. CT was performed with a 3-5-mm slice thickness of the head and neck region. For patients treated with 3DCRT, the initial treated target volume was the gross target volume with a 2-cm margin in all directions, and shrinkage to avoid excessive irradiation to the pons and spinal cord after 46 Gy. All patients were treated with bilateral opposing portals to cover the primary tumor and the neck; the fraction size was 2 Gy. After 36 Gy, the primary tumor and the neck were treated with a split-field technique. Regarding shrinkage, the primary tumor was irradiated with bilateral opposing fields, using 2.5 Gy as the fraction size; an additional 10 Gy was administered. An additional 24 Gy in 10 fractions was delivered to the nasopharynx via bilateral anterior oblique infraorbital portals. The accumulated radiation dose to the nasopharynx was 70 Gy in 32 fractions. For patients with N0 to N3a disease, the neck was further treated by using anterior-posterior opposing portals to administer 36 Gy in 18 fractions, with the spinal cord shielded. The accumulated dose was 50 Gy in 25 fractions to the uninvolved neck and 60 Gy in 30 fractions to the involved regions. After 60 Gy, an additional 5 Gy in 2 fractions was administered to the residual neck masses. 3DCRT was delivered using the 6-MV linear accelerator Siemens Mevatron (Siemens AG, Berlin, Germany).

**Figure 3:**
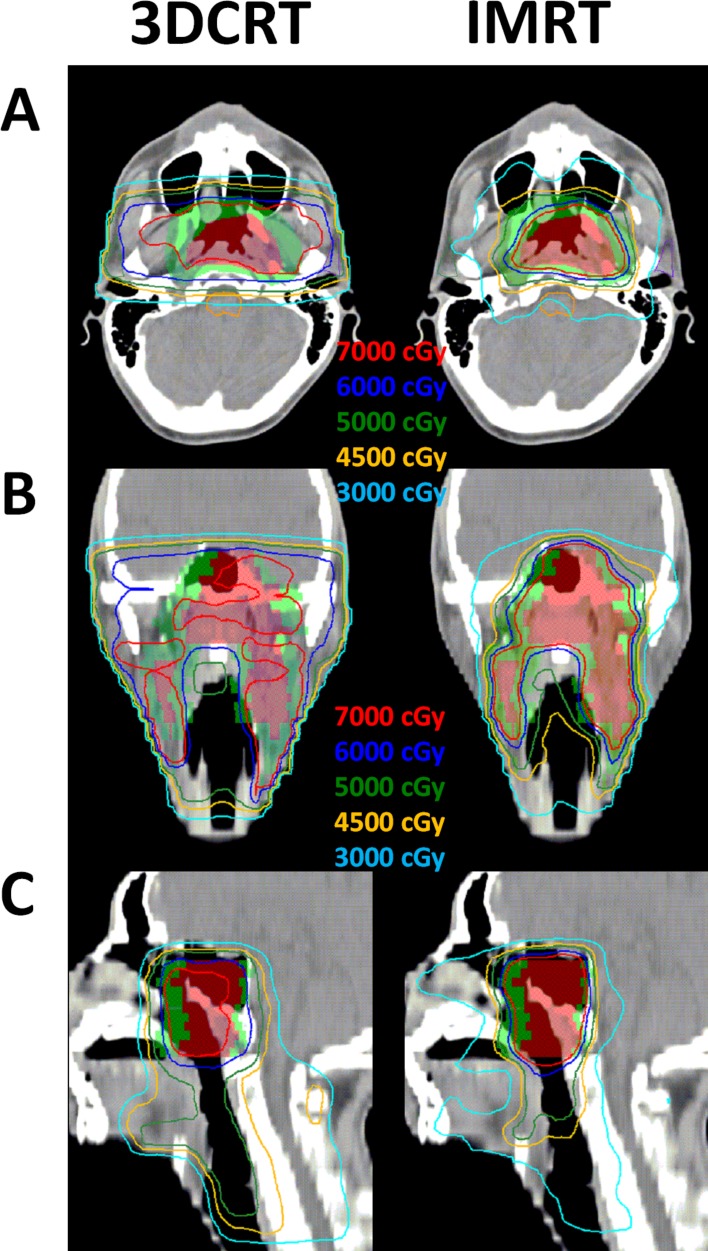
Isodose distributions in one NPC patient The isodose distributions for one NPC patient (cT3N2M0) planned by 3DCRT (left) and IMRT (right) displayed on the **A**. axial, **B**. coronal, and **C**. sagittal planes. Color-wash areas: CTV-70: red; CTV-50: green. The red, blue, green, orange, and indigo lines were isodose curves of 70 Gy, 60 Gy, 50 Gy, 45 Gy, and 30 Gy.

For patients treated with IMRT, the target areas received the following 3 dose levels with simultaneous integrated boosts, in accordance with the RTOG 0225 protocol [2]: clinical target volume (CTV)-70 (70 Gy for the gross nasopharyngeal tumor and lymphadenopathy); CTV-63/60 (60-63 Gy for subclinical disease and high-risk lymphatic regions, including the entire nasopharynx, retropharyngeal nodal regions, skull base, clivus, pterygoid fossae, parapharyngeal space, sphenoid sinus, and posterior nasal cavity/maxillary sinuses that include the pterygopalatine fossa and upper middle neck regions; a concentric volume that completely encompassed the entire CTV-70 in all directions); and CTV-56/54 (54-56 Gy to the low-risk regions, including the low/middle neck and supraclavicular fossa regions). The planning target volumes were evenly expanded using a 4-mm margin. Two dose fractionations (33 or 35 fractions) were used, at the discretion of the treating physician. The plans were optimized using an inverse planning algorithm (Direct Machine Parameter Optimization) and heterogeneity corrections. The isodose distributions for NPC patients planned by 3DCRT and IMRT are demonstrated in Figure 3. IMRT was delivered using an Elekta Synergy accelerator (Elekta, Stockholm, Sweden) with a step-and-shoot technique. The treatment position was verified weekly by using cone-beam CT X-ray volume imaging.

### Chemotherapy

Concurrent chemoradiotherapy regimens included 30 mg/m^2^ cisplatin weekly during radiotherapy or 100 mg/m^2^ cisplatin every 3 weeks for 2-3 cycles, at the discretion of the chemo-oncologist. Neoadjuvant chemotherapy was administered mainly for patients with obvious intracranial invasion, supraclavicular or bilateral neck lymph node metastasis, or large neck nodes (>6 cm) [1], at the discretion of the chemo-oncologist. The induction chemotherapy regimens were MEPFL (8 mg/m^2^ mitomycin, 60 mg/m^2^ epirubicin, and 60 mg/m^2^ cisplatin on day 1, and 450 mg/m^2^ fluorouracil and 30 mg/m^2^ leucovorin on day 8) or PF (100 mg/m^2^ cisplatin on day 1 and 1000 mg/m^2^ fluorouracil on days 1-3) every 3 weeks for 2 or 3 cycles. None of the patients received adjuvant chemotherapy.

### Follow-up assessment

Acute toxicities were rated according to the Radiation Therapy Oncology Group (RTOG) criteria [19]. All patients were followed-up every 2-3 months for the first 2 years, every 4 months for the third year, and every 6 months after the third year until recurrence or death. After completion of treatment, the treatment response after radiotherapy was assessed every 3 months by using endoscopy and head and neck MRI as well as a biopsy of the nasopharynx if suspicions of recurrence were identified. Chest radiographs were taken every 6 months, whereas CT, MRI, bone scanning, or other investigations were performed when clinical suspicions of recurrence were identified. Late toxicities were assessed according to the late morbidity scoring criteria of RTOG.

### Statistical analysis

Statistical analysis was performed using the Statistical Package for Social Sciences for Windows, version 17.0 (SPSS, Chicago, IL). Survival data were confirmed with the Cancer Registry Medical Information Management Office in our hospital. All events were calculated from the date of treatment completion. To obtain equivalent follow-up times for both groups, an analysis was conducted using the follow-up data available on June 20, 2015 for 3DCRT patients and on July 30, 2016 for IMRT patients. Actuarial estimates of LRC, distant metastasis-free survival (DMFS), and overall survival (OS) were calculated using the Kaplan-Meier method and compared using the log rank test. The log-rank test was used to determine the prognostic factors affecting survival. All prognostic variables found to be significant in univariate analysis were included in multivariate analysis using the Cox proportional hazards regression model. A *p*-value of ≤0.05 was considered statistically significant.
